# Correlation of TROP-2 expression with clinical–pathological characteristics and outcome in triple-negative breast cancer

**DOI:** 10.1038/s41598-022-27093-y

**Published:** 2022-12-28

**Authors:** Hava Izci, Kevin Punie, Lise Waumans, Annouschka Laenen, Hans Wildiers, Freija Verdoodt, Christine Desmedt, Jan Ardui, Ann Smeets, Sileny N. Han, Ines Nevelsteen, Patrick Neven, Giuseppe Floris

**Affiliations:** 1 Department of Oncology, KU Leuven, Herestraat 49, Box 7003-06, 3000 Leuven, Belgium; 2 Department of General Medical Oncology, University Hospitals Leuven, Leuven, Belgium; 3 Department of Imaging & Pathology, University Hospitals Leuven, Leuven, Belgium; 4 Leuven Biostatistics and Statistical Bioinformatics Centre, KU Leuven, Leuven, Belgium; 5 Research Department, Belgian Cancer Registry, Brussels, Belgium; 6 Department of Surgical Oncology, University Hospitals Leuven, Leuven, Belgium; 7 Department of Gynecological Oncology, University Hospitals Leuven, Leuven, Belgium

**Keywords:** Cancer, Breast cancer

## Abstract

Limited data exist regarding the associations between TROP-2 protein expression, clinical–pathological characteristics, and outcome in triple-negative breast cancer (TNBC). TROP-2 expression was determined for patients diagnosed with TNBC between 2000 and 2017 by immunohistochemistry (IHC) (ab227689, Abcam) on whole slide tumor sections, and assessed as continuous and categorical variables (H-score high, 201–300, medium 100–200 and low < 100). We investigated the prognostic value of TROP-2 expression for relapse and survival, associations between TROP-2 expression and baseline patient and tumor characteristics, stromal tumor-infiltrating lymphocytes (sTILs), androgen receptor (AR), standardized mitotic index (SMI) and pathological complete response (pCR, in patients with neoadjuvant chemotherapy) were assessed. We included 685 patients with a median age at diagnosis of 54 years (range 22–90 years). After median follow-up of 9.6 years, 17.5% of patients experienced distant relapse. TROP-2 expression was high, medium and low in 97 (16.5%), 149 (25.3%) and 343 (58.2%) of patients, respectively. The presence of LVI, associated DCIS, nodal involvement, apocrine histology and AR expression were correlated with higher TROP-2 levels. There were no associations between TROP-2 expression and sTILs, time-to-event outcomes, or pCR rate after neoadjuvant chemotherapy. TROP-2 expression is not associated with sTILs level and has no prognostic value in our cohort of stage 1–3 TNBC. However, an association with histotype and AR expression was found, suggesting a histotype specific TROP-2 expression pattern with highest expression in apocrine subtype, warranting further research.

## Introduction

Patients with triple-negative breast cancer (TNBC) account for 10–15% of all breast cancer cases^1^. Their tumors lack both expression of estrogen and progesterone receptors and overexpression of the human epithelial growth factor (HER2) receptor, rendering them non-eligible for endocrine or traditional HER2-targeted therapy. These cancers generally have a worse prognosis and higher recurrence rates within the first 3 years after diagnosis when compared to the other subtypes. The identification of reliable prognostic and predictive factors in TNBC remains an unmet medical need.

TNBC is an exclusion diagnosis, consisting of a heterogeneous breast cancer subtype with variable morphology and biology. Despite this, there is limited impact of further biological differentiation on current treatment patterns for TNBC^2^. The intrinsic molecular subtype classifications by Lehmann et al. and Burstein et al. have characterized luminal androgen receptor (LAR), mesenchymal, immunomodulatory and basal subtypes on the transcriptomic level^3–5^. In some cases, the histological subtype can infer molecular subtype: e.g., apocrine carcinomas are often in the LAR group, while metaplastic carcinomas fit in the mesenchymal group and breast carcinomas with medullary features (BC^medullary^) likely fall within the immunomodulatory group^6–9^. Each of these subtypes have different prognoses and may require different therapeutic strategies. As each subtype has a different response to treatment and clinical outcome, tailored treatment for patients with TNBC is fundamental.

Androgen receptor (AR), which is expressed in 10–50% of TNBC, can stimulate tumor cell growth in TNBC^10,11^. Stromal tumor infiltrating lymphocytes (sTILs), which can be considered surrogate markers for an anti-tumor immune response, are more prevalent in TNBC compared to other breast cancer subtypes^12^. sTILs have been shown to provide robust prognostic value in early and loco-regionally advanced TNBC, both in patients with and without (neo) adjuvant chemotherapy, and strongly correlate with pathological complete response (pCR) rates after neoadjuvant chemotherapy (NACT)^12–18^.

Antibody–drug conjugates (ADCs) have emerged as a new promising treatment option for solid tumors. In the treatment landscape of advanced TNBC, sacituzumab govitecan (SG) is now approved by the United States Food and Drug Administration and the European Medicine Agency for the treatment of advanced TNBC after 2 prior regimens, of which at least one for metastatic disease. SG is an ADC targeting TROP-2 (trophoblast cell-surface antigen-2), linked by a cleavable linker to SN38, a topoisomerase-1 inhibitor and the active metabolite of Irinotecan^19^. TROP-2 is an interesting but poorly explored tumor-associated cell-surface antigen, which has been associated with increased tumor aggressiveness and metastatic potential when overexpressed in cancer cells^20–22^. TROP-2, a transmembrane calcium protein belonging to the EpCAM (epithelial cell adhesion molecule) family, is encoded by the tumor-associated calcium signal transducer 2 (*TACSTD2*) gene on chromosome 1p32. TROP-2 is expressed at high levels by normal human multistratified epithelia and trophoblast cells. Overexpression can be present in several solid tumors, including TNBC. The precise role of TROP-2 in invasion and metastasis is poorly understood, but seems to differ between different cancer types and be modulated by different pathways^23^.

TROP-2 expression has been investigated to a limited extend in breast cancer regardless of subtype and more specifically in patients with advanced TNBC^24^. The characteristics and prognostic value of this marker have not been thoroughly evaluated in patients with early and locally advanced TNBC. Therefore, our aim was to evaluate the expression of TROP-2 in early and locally advanced TNBC (primary endpoint) and to investigate potential associations with clinical–pathological characteristics and survival outcomes (secondary endpoints).

## Methods

### Patients

**Figure 1:**
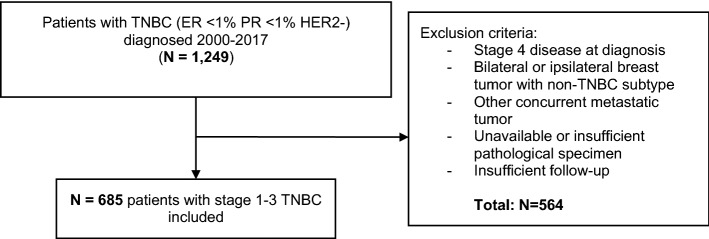
Study population flow diagram.

Patients with primary diagnosis of stage 1–3 TNBC between 1st January 2000 and 31st December 2017 at University Hospitals Leuven were included. Selection for the presented analysis required availability of information on TROP-2 expression, sTILs, and AR immunohistochemistry (IHC). We excluded patients with metastatic disease at time of diagnosis, bilateral or ipsilateral tumor with non-TNBC phenotype, metachronous non-TNBC tumor, other concurrent metastasized tumors as well as patients that were lost during follow-up (minimum follow-up 4 m) or for which the surgery was performed in another hospital and specimen was not available in our biobank (Fig. 1).

Patient and tumor characteristics were retrieved from the clinical database and the medical records (age, body mass index (BMI), family history, menopausal status, tumor size, histological subtype, grade of differentiation, section margins, presence of DCIS, lymphovascular invasion (LVI), lymph node involvement, pCR, data on relapse, survival and cause of death).

### Histology

A 5 µm thick freshly cut slide of formalin-fixed, paraffin-embedded (FFPE) tumor blocks was used to review the tumor type on H&E according to recent literature, measure sTILs and assess Standardized Mitotic Index (SMI). Two pathologists (G.F. and L.W.) scored sTILs on H&E slides according to the recommendations by the international sTILs working group, in core needle biopsies pre-neoadjuvant chemotherapy and in the resection specimens of patients treated with up-front surgery^25^. The intra-class correlation coefficient and Bland–Altman analysis was done to assess the inter-rater variability. Low sTILs were defined as < 30%, intermediate 30–49% and high as ≥ 50%^13,12^.

SMI was counted manually on a representative H&E section by two pathologists only in the resection specimens of patients treated with up-front surgery (L.W. and G.F.), according to the publication of Collan et al.^26^. SMI was assessed with a correction for the proportion of tumor cell nests expressed as % of the area occupied for each microscopic field according to Haapasalo et al.^27^. The median SMI value was used as cut-off to distinguish high-SMI from low-SMI tumors.

We then assessed protein-level expression of TROP-2 and AR by IHC on the Bond Automatic IHC Stainer (Leica Biosystems). A 1:150 dilution of rabbit monoclonal anti-TROP-2 antibody (Ab227689 by Abcam) was used, pre-treated for 20 min at pH 9, and incubated for 30 min. Expression of TROP-2 antibody was scored semiquantitatively using the H-score. We subdivided the scores into categories: low scores < 100, medium scores 100–200, and high scores 201–300. A 1:100 dilution of monoclonal mouse anti-human AR (clone AR411, DAKO), was used. Two pathologists (L.W. and G.F.) scored the stains, using both the percentage of positive cells (0–100%) and the intensity of the nuclear staining pattern (0–3). For statistical analysis, we used both 1% and 10% as cut-off scores for positivity.

### Objectives and endpoints

The primary objective of this study was to evaluate qualitative and semi-quantitative characteristics of TROP-2 expression in early and locally advanced TNBC using descriptive analysis with H-scores and spatial distribution of TROP-2 expression. Secondary objectives were to investigate potential associations between TROP-2 expression, sTILs and SMI and (1) clinical–pathological characteristics (age, BMI, tumor grade and size, LVI, nodal involvement, DCIS, histology, pCR rate), and (2) time-to-event outcomes (breast cancer-specific survival (BCSS), distant recurrence-free interval (DRFI), and invasive disease-free survival (IDFS)).

### Statistical analysis

Associations between continuous variables were assessed by the Spearman correlation coefficient, and group differences by the Kruskal–Wallis test for multiple groups or Mann–Whitney U test for two groups. Associations between categorical variables were assessed by means of the Fisher exact test.

BCSS and DRFI were estimated using the cumulative incidence function and IDFS using Kaplan–Meier estimates^28^. BCSS is defined as the time between diagnosis and death of breast cancer. Death of other causes is considered as a competing event. Patients alive are censored at last follow-up. DRFI is defined as the time between diagnosis and metastasis or death of breast cancer. Death of other causes is considered as a competing event. Patients alive without metastasis are censored at last follow-up. IDFS is defined as the time between diagnosis and any relapse or death of any cause. Patients alive without relapse are censored at last follow-up.

Cox proportional hazards models were used to assess the association between TROP-2 expression and outcome. Results are presented as hazard ratios (HR) with 95% confidence intervals. Analyses were performed using SAS software (version 9.4 of the SAS System for Windows).

### Ethics approval and consent to participate

This study involving human participants was in accordance with the 1964 Helsinki Declaration and its later amendments or comparable ethical standards. The Ethics Committee (IRB) of University Hospitals Leuven approved this study. Informed consent for use of tissue samples of all human participants was obtained.

## Results

### Patients

A total of 685 patients were included for which TROP-2, AR-IHC and sTILs were available. Median age at diagnosis was 54 years (range 22–90 years) (Table 1). As expected, most of the tumors in our cohort were invasive breast carcinoma of no special type (IBC-NST) (79.6%), while other histological subtypes were less frequent: BC^medullary^ (5.8%), metaplastic carcinoma (5.0%), apocrine carcinoma (4.5%), other histology (3.2%), or mixed tumors (1.9%). Lymph node involvement and LVI was present in 36.2% and 23.4% of patients, respectively, while associated DCIS was observed in 62.8% of patients.

**Table 1:** Baseline patient- and tumor characteristics for all patients.

Variable	Statistic	Total
N	N	685
Age (year)	Median	54.0
	Range	(22.0; 90.0)
**Menopausal status**		
Pre/perimenopause	n (%)	274 (40.9%)
Postmenopause	n (%)	396 (59.1%)
NA	n	15
BMI (kg/m^2^)	Median	24.7
	Range	(14.2; 48.1)
**T-stage**		
T1	n (%)	288 (42.0%)
T2		330 (48.2%)
T3		44 (6.4%)
T4		23 (3.4%)
NA		0
**N-stage**		
N0	n (%)	436 (63.8%)
N1		180 (26.4%)
N2		41 (6.0%)
N3		26 (3.8%)
NA		2
**N-stage**		
Negative	n (%)	436 (63.8%)
Positive		247 (36.2%)
NA		2
**Grade**		
1	n (%)	9 (1.3%)
2		70 (10.2%)
3		606 (88.5%)
NA		0
**DCIS**		
No	n (%)	255 (37.2%)
Yes		430 (62.8%)
NA		0
**LVI**		
No	n (%)	428 (76.6%)
Yes		131 (23.4%)
NA		126
**Histology**		
IBC-NST	n (%)	545 (79.6%)
Mixed		13 (1.9%)
Apocrine		31 (4.5%)
BC^medullary^		40 (5.8%)
Metaplastic		34 (5.0%)
Other*		22 (3.2%)
NA		0

*NA* not available, *CT* chemotherapy, *DCIS* ductal carcinoma in situ, *LVI* lymphovascular invasion, *IBC-NST* invasive breast carcinoma of no special type.

*Other histology (N = 22): N = 6 invasive lobular carcinoma (ILC), N = 4 Pleomorphic ILC N = 4 adenoid cystic, N = 2 micropapillary, N = 1 mucinous, N = 1 invasive papillary, N = 1 secretory, N = 1 polymorphous carcinoma N = 1 myoepithelial carcinoma, N = 1 neuroendocrine carcinoma, small cell.

After a median follow-up of 9.6 years, 33 loco-regional events and 112 distant relapse events, were observed. Breast cancer-related deaths occurred in 16.2% of patients (103 events), with death due to other causes recorded in 8.8% of patients. Estimates for 2-, 5-, and 10-year IDFS, DRFI and BCSS are available in Supplementary Table S1.

### Histopathological assessment of biomarkers

**Figure 2:**
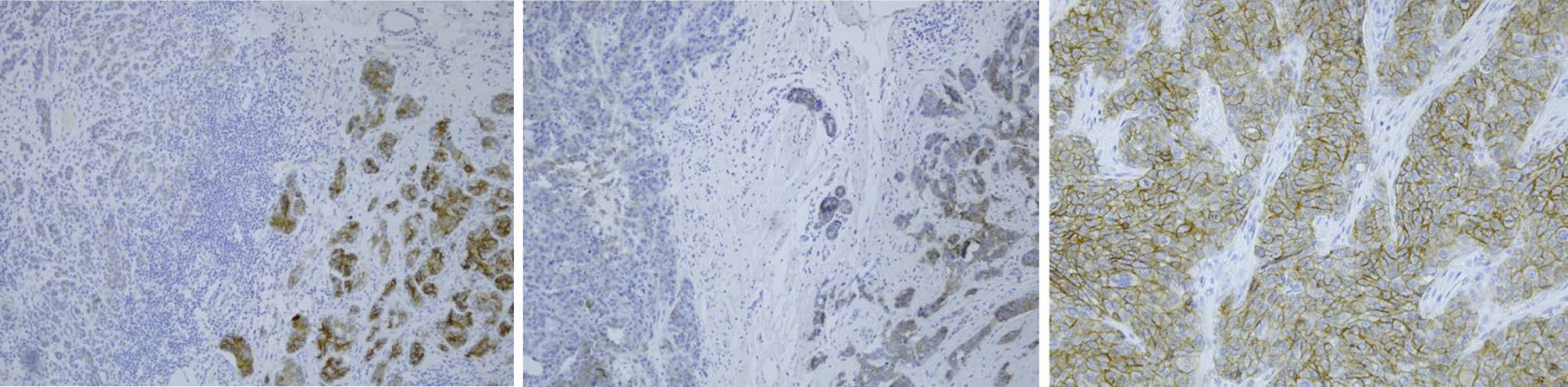
Expression of TROP-2 on pathological specimen. Intermediate expression of Trop-2 (left and middle), and high expression of TROP-2 (right). Positivity of TROP-2 was cytoplasmic, membranous, or both. Intermediate to low TROP-2 expression showed heterogeneous staining pattern as shown in the left and middle panel. Immunohistochemistry was scored by using the semiquantitative H-score method to better capture this heterogeneity in the lower expression range.

TROP-2 staining showed membranous positivity, which in most cases was variable in intensity throughout the tumor (Fig. 2). In some cases, associated DCIS showed stronger expression of TROP-2 than the invasive carcinoma. Positive internal control was present in normal ductulo-lobular units and was rather weak in intensity. The mean H-score was 88.0 (range 0–300). Of the 589 patients with TROP-2 staining available, expression was high (H-score 201–300), medium (H-score 100–200) and low (H-score < 100) in 97 (16.5%), 149 (25.3%) and 343 (58.2%) patients, respectively (Table 2). Notably, 151 (25.6%) samples had absent or extremely low staining (H-score 0–10).

**Table 2:** TROP-2 expression, AR expression, sTILs and SMI.

Variable	Statistic	Total
TROP-2 score	N	589
	Mean	88.0
	Median	70.0
	Range	(0.0; 300.0)
**TROP-2 categorical**		
Low H-score < 100	n (%)	343 (58.2%)
Medium H-score 100–200		149 (25.3%)
High H-score 201–300		97 (16.50%)
NA		96
AR percentage	N	602
	Mean	14.9
	Median	0.0
	Range	(0.0; 100.0)
**AR (1% cutoff)**		
Negative	n (%)	412 (68.1%)
Positive		193 (31.9%)
NA		80
**AR (10% cutoff)**		
Negative	n (%)	457 (75.5%)
Positive		148 (24.5%)
NA		
sTILs score	N	606
	Mean	26.5
	Median	20.0
	Range	(0.0; 96.0)
**sTILs categorical**		
Low (> 30)	n (%)	381 (62.9%)
Medium (30–49)		109 (18.0%)
High (> 50)		116 (19.1%)
NA		79
SMI	N	457
	Mean	19.3
	Median	16.8
	Range	(0.0; 128.5)
**SMI (binary)**		
< Median (16.8)	n (%)	229 (50.1%)
≥ Median (16.8)		228 (49. 9%)
NA		228

*AR* androgen receptor, *sTILs* stromal tumor-infiltrating lymphocytes, *SMI* standardized mitotic index, *NA* not available.

With a 10%-cutoff, 148 (24.5%) of the cases were considered AR-positive, while based on the 1% cutoff, AR-positivity was observed in 193 cases (31.9%). All apocrine carcinomas were strongly AR-positive as expected (Table 4).

Low, intermediate and high sTILs were observed in 62.9%, 18% and 19.1%, with a median score of 20 (range 0–96) (Table 2). The inter-rater reliability defined by intra-class correlation coefficient was 0.87 (95% C.I. 0.73–0.94) in 100 samples. We observed no significant difference between raters from the Bland–Altman analysis (p = 0.5).

The median SMI was 16.8. Using this median threshold, 49.9% of patients were classified as high SMI, while 50.1% had a low SMI.

### Associations between TROP-2 expression and clinical–pathological characteristics

**Figure 3:**
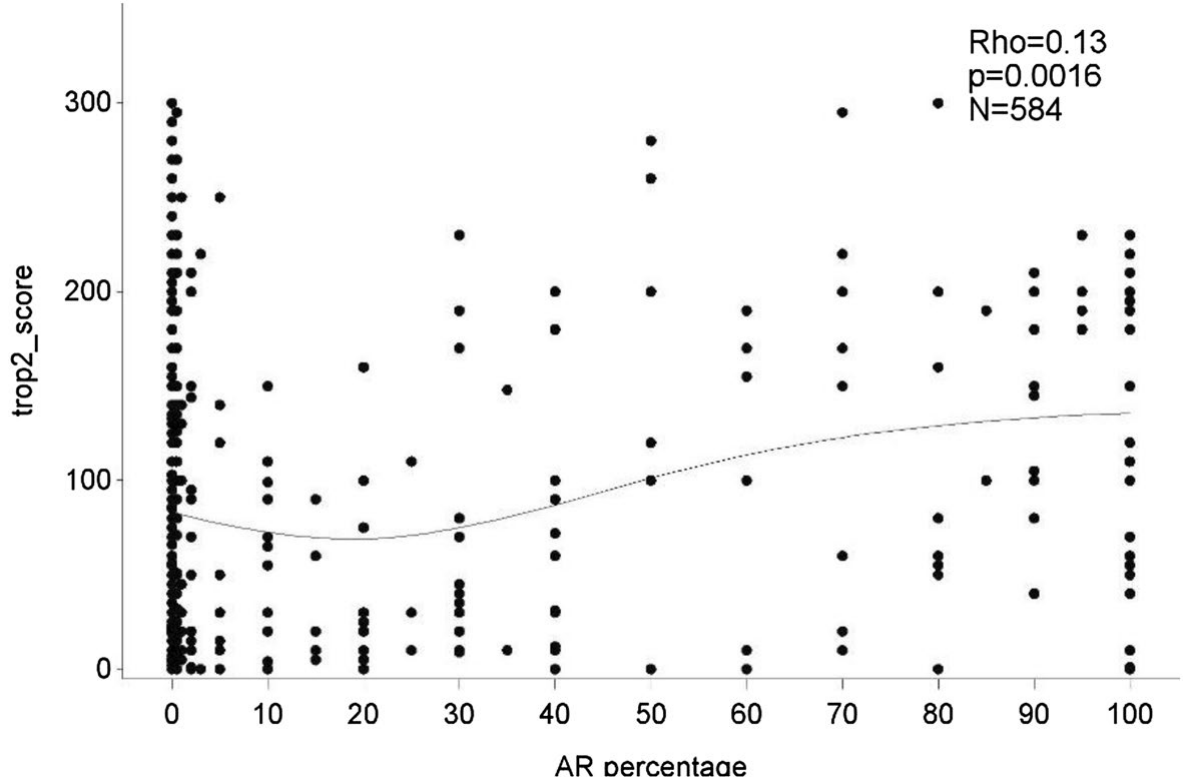
TROP-2 expression score was positively correlated with AR expression (10% cutoff). N = 584, ρ = 0.13, p = 0.002.

Higher TROP-2 expression was correlated with the presence of LVI (p = 0.006) and DCIS (p < 0.001), both as a continuous and categorical variable. TROP-2 expression was significantly associated with lymph node involvement when evaluated as a continuous score (p = 0.02) and a similar trend was observed for the categorical score. Higher TROP-2 expression was associated with higher continuous and categorical AR expression with a 10%-cutoff (continuous: ρ = 0.13, p = 0.002, categorical: p = 0.009) (Fig. 3). No significant correlations were observed between TROP-2 expression and tumor size, grade, sTILs or SMI. Continuous higher TROP-2 expression showed a correlation with lower continuous BMI (ρ = − 0.09, p = 0.03); however, this was not significant when BMI was evaluated as a categorical variable (Tables 3, 4). There was a significant interaction between histological subtype and TROP-2 expression (global test p < 0.001). All apocrine carcinomas showed an intermediate or high TROP-2 score with median H-score of 175.0, which was significantly higher compared to other subtypes (vs IBC-NST median H score 70.0 p < 0.001, vs medullary median 40.5 p = 0.01, vs metaplastic median 45.0 p < 0.001, vs other median 15.0 p < 0.001).

**Table 3:** Univariable analyses of interactions between continuous TROP-2 expression and continuous clinical–pathological variables.

Association of TROP-2 score with	Correlation statistics	Spearman correlation (ρ	95% confidence interval	p-value
sTILs	Spearman	0.018	(− 0.063; 0.098)	0.6713
AR expression	Spearman	0.130	(0.050; 0.209)	0.0016
Standardized mitotic index	Spearman	0.013	(− 0.080; 0.105)	0.7861
Age	Spearman	− 0.025	(− 0.106; 0.056)	0.5450
BMI	Spearman	− 0.090	(− 0.169; − 0.009)	0.0299

*AR* androgen receptor, *sTILs* stromal tumor-infiltrating lymphocytes.

**Table 4:** Univariable analyses of interactions between continuous TROP-2 expression and categorical clinical–pathological factors.

Variable	Category	N	Median TROP-2 expression H-score	IQR TROP-2 expression H-score	p-value
AR expression (1% cutoff)	AR positive	187	80.8	(20.0–180.0)	0.070
	AR negative	398	81.4	(10.0–150.0)	
AR expression (10% cutoff)	AR positive	146	102.0	(20.0–180.0)	0.009
	AR negative	439	55.0	(10.0–140.0)	
Tumor size (TNM)	T1	266	81.0	(15.0–155.0)	0.598
	T2	260	82.0	(10.0–155.0)	
	T3	40	87.2	(4.5–145.0)	
	T4	23	62.0	(20.0–120.0)	
Nodal status	Node positive	376	80.0	(20.0–180.0)	0.016
	Node negative	211	60.0	(10.0–149.0)	
Nodal status (TNM)	N0	376	60.0	(10.0–149.0)	0.119
	N1	155	75.0	(20.0–180.0)	
	N2	32	88.0	(27.5–160.0)	
	N3	24	95.0	(20.0–165.0)	
Differentiation grade	1	7	120.0	(0.0–150.0)	0.479
	2	60	100.0	(20.0–165.0)	
	2	522	60.0	(10.0–150.0)	
Associated DCIS	Absent	221	40.0	(7.0–125.0)	< 0.001
	Present	368	82.5	(20.0–180.0)	
LVI	Absent	359	70.0	(20.0–150.0)	0.006
	Present	111	100.0	(30.0–200.0)	
Histological subtype	IBC-NST	471	70.0	(12.0–150.0)	< 0.001
	Mixed	11	70.0	(20.0–150.0)	
	Apocrine	26	180.0	(110.0–200.0)	
	BC^medullary^	34	40.5	(7.0–150.0)	
	Metaplastic	27	45.0	(10.0–100.0)	
	Other	20	15.0	(0.0–95.0)	

*IQR* inter-quartile range, *AR* androgen receptor, *DCIS* ductal carcinoma in situ, *LVI* lymphovascular invasion, *IBC-NST* invasive breast carcinoma of no special type.

### Associations between TROP-2 expression and outcome

When evaluating TROP-2 expression as a continuous variable, we detected no significant differences in long-term time-to-event outcomes according to TROP-2 expression, although a trend towards improved IDFS, DRFI and BCSS for higher TROP-2 expression was observed. After adjustment for age, nodal status, tumor stage, grade, and continuous AR expression, the trend became significant for IDFS (HR 0.91 (95% C.I. 0.96–1.00); p = 0.049), but not for BCSS and DRFI. TROP-2 expression as a categorical variable was not significantly associated with the evaluated time-to-event outcomes in univariable or multivariable analyses (Table 5).

**Table 5:** Univariable and multivariable analyses of prognostic value of TROP-2 expression (continuous and categorical).

Distribution TROP-2 expression	Analysis	Outcome	Hazard ratio (95% CI)	p-value
Continuous (+ 10 units H-score)	Univariable	BCSS	0.978 (0.953;1.003)	0.089
		DRFI	0.978 (0.954;1.002)	0.075
		IDFS	0.982 (0.964;1.000)	0.051
	Multivariable	BCSS	0.983 (0.957;1.009)	0.205
		DRFI	0.982 (0.958;1.007)	0.165
		IDFS	0.981 (0.963;1.000)	0.049
Categorical (global test)	Univariable	BCSS	NA	0.434
		DRFI		0.415
		IDFS		0.235
	Multivariable	BCSS		0.433
		DRFI		0.472
		IDFS		0.264

*HR* hazard ratio, *CI* confidence interval, *HR* > *(*<*)1* increased(decreased) risk with increasing TROP2 level, corrected for: age, N-positive, T-stage, grade, AR percentage, *NA* not available.

TROP-2 was not associated with pCR, which was observed in 22 out of 64 patients treated with NACT. There was no significant difference in TROP-2 expression when we compared expression on CNB before NACT with expression on residual invasive cancer tissue following NACT and surgery (p = 0.618).

### Associations between sTILs, SMI, clinical–pathological characteristics and outcome

Higher sTILs were associated with higher SMI (ρ = 0.19, p < 0.001), younger age (ρ =  − 0.14, p < 0.001), lower BMI (ρ =  − 0.1, p = 0.01), lower AR percentage (ρ =  − 0.09, p = 0.03), smaller tumor size (p < 0.001), higher tumor grade (p < 0.001) and absence of DCIS (p < 0.001). The highest TILs were observed in BC^medullary^ (median 64), and lowest in the apocrine subtype (median 6) (global test p < 0.001).

Higher SMI was not correlated with outcome, but correlated with continuous TILs (ρ = 0.19, p < 0.001), higher grade (p < 0.001), and with decreasing continuous AR expression (ρ =  − 0.158, p < 0.001), age (ρ =  − 0.25, p < 0.001) and BMI (ρ =  − 0.10, p =  0.04).

## Discussion

TROP-2 is an emerging biomarker which has raised therapeutic interest as target for ADCs, among which SG, that is currently approved for advanced TNBC and being studied in a variety of cancer types. We investigated semiquantitative TROP-2 expression in patients with early and loco-regionally advanced TNBC treated in an academic hospital and correlated this with demographics and clinical–pathological characteristics, including sTILs, AR expression, histological subtype, and long-term outcomes.

We observed TROP-2 expression in about 86% (n = 589/685) of the cases in our cohort. This large proportion of TROP-2-positivity is consistent with the currently reported literature^20^. Previous studies have shown higher levels of TROP-2 protein and gene (over) expression in TNBCs (88%) compared to other cancer types or other subtypes of breast cancer^20,23,29^. However, in advanced TNBC, the proportion of patients with high and medium TROP-2 expression according to the biomarker analysis from ASCENT was considerably higher when compared to those in our series^24^. This can be explained by differences in sensitivity and specificity of the different antibodies used in both studies and by potential differences in TROP-2 expression between the early and advanced TNBC settings.

A previous study by Ambrogi et al. in early breast cancers has shown a worse prognosis in cases with higher membranous expression of TROP-2^20^. Our study did not demonstrate significant associations between TROP-2 expression and time-to-event outcomes and could as such not confirm the negative prognostic value of TROP-2 expression within stage 1–3 TNBC. We even observed a trend towards improved survival with higher TROP-2 expression (evaluated as continuous variable). It is unclear how this favorable prognostic trend should be interpreted, taking into account the association between TROP-2 expression and negative baseline prognostic factors such as lymph node or lymphovascular involvement in our cohort. A limited number of events warrant caution in the interpretation of the prognostic value of TROP-2 expression. Ambrogi et al. demonstrated differential prognostic value of membranous and cytoplasmic expression using two different antibodies, with cytoplasmic expression as favorable prognostic biomarker. The antibody used in our study stains both membranous and cytoplasmic TROP-2, which can potentially explain the absence of prognostic value for TROP-2 expression in our study.

The application of TROP-2 as a target for ADCs has already been clinically validated considering the successful trial results and approval of SG for patients with advanced TNBC^19^. Uncertainty remains regarding the role of TROP-2 IHC as predictive biomarker for the benefit of SG. The biomarker analysis of ASCENT showed improved outcomes for patients with SG compared to treatment of physician’s choice in patients with high, medium and low TROP-2 expression. However, the numerical increase in outcome seemed higher in groups with high and medium TROP-2 expression^24^. Caution in interpretation is warranted, given small numbers precluded formal testing. Additional studies are needed to assess whether higher TROP-2 expression is predictive for better response to SG in advanced TNBC.

Our study showed a correlation of high TROP-2 expression with the presence of LVI and presence of nodal involvement both on the continuous and categorical TROP-2 assessment. TROP-2 has been described to be involved in the PI3K/AKT pathway, which induces epithelial-mesenchymal transition (EMT)^23,29,30^. Since metaplastic carcinomas show a high grade of EMT, we anticipated higher TROP-2 scores in this subtype. In all metaplastic carcinomas in our series, we found significantly lower TROP-2 expression compared to other subtypes. This suggests that TROP-2 overexpression is unlikely to be associated with EMT in primary early and loco-regionally advanced TNBC. Other studies have also found a possible role in angiogenesis^23,31^, which could potentially explain our observed association between LVI and high TROP-2 expression.

We observed AR-positivity in 31.9% of cases with a 1%-cutoff and 24.5% with a 10%-cutoff. These results are in line with previous studies, which suggest AR-positivity in 10–50% of TNBC^11^. All apocrine carcinomas with known AR status in our cohort were strongly AR-positive. Strong AR-positivity is typical for apocrine carcinomas, making this a good surrogate for the LAR subtype^32^. Additionally, apocrine tumors and non-apocrine tumors with higher AR expression showed significantly higher TROP-2 expression, which could suggest TROP-2 is overexpressed in the LAR subtype. Recent studies in prostate cancer have also shown a co-expression of TROP-2 and AR^33,34^. TNBCs from the LAR subtype are known to be enriched in *PIK3CA, AKT1* and *CDH1* mutations, which could also explain the association between TROP-2 and AR expression^35^. In line with previous studies, we also found AR linked to a lower SMI and higher age in our cohort. AR-negative tumors are more likely to show higher mitotic activity and presumably more aggressive clinical behavior. Previous studies have shown a lower proliferation on Ki-67 and lower mitotic activity in AR-positive tumors^32,36^. A lower response to chemotherapy in the LAR group could possibly be due to these tumors being less mitotically active^32^. Despite correlation between TROP-2 and AR expression, TROP-2 expression was not associated with SMI or grade but was significantly correlated with LVI and nodal involvement, which could indicate a surrogate factor for lymphatic spreading of the metastases. This could be of potential value in tailored treatment and new combination therapies of TROP-2-targeted treatment, AR-targeting agents and other anticancer therapies.

We found high sTILs with a cut-off of 30% in 19.1% of cases. This is in line with literature, which suggests high sTILs in 4–37% of TNBC^37,38^. We could also confirm previous studies showing a higher number of sTILs in patients with lower age and lower BMI^32,39^. We did not find any correlation of TROP-2 with sTILs in our cohort. This lack of correlation might be in part related to the fact that immuno-modulatory infiltrates may be variably distributed across the diverse TNBC histotypes and molecular subtypes, as indicated by the work of Gruosso et al., and by the revised molecular classification by Lehmann et al.^40,41^. Further research is needed to understand to which extent TROP-2 expression is related to specific patterns of immune-modulatory infiltrates or specific subtypes of inflammatory cells. We did find an excellent intra-class correlation coefficient or agreement between the two pathologists scoring sTILs in this study.

One of the strengths of our study is that we used a uniform cohort of patients with stage I–III TNBC, which allowed us to investigate the prognostic value and association with baseline characteristics in this setting. We also had a long follow-up time available for most included patients.

Limitations of our study were the retrospective design and the relatively limited sample size with small number of events, which requires caution in interpretation of prognostic analysis. At present, no standardized and internationally accepted guidelines are available for TROP-2 IHC.

In our patient cohort with early and loco-regionally advanced TNBC, higher TROP-2 expression was associated with apocrine histology, higher AR expression, presence of DCIS, LVI and nodal involvement. Additional research is necessary to confirm our association between baseline characteristics and TROP-2 expression. There was no correlation between TROP-2 expression and sTILs or outcome, but limited numbers warrant caution in interpretation, and the prognostic value of TROP-2 expression in early TNBC remains to be further investigated. With the emergence of TROP-2-directed ADCs such as SG and the potential transition of these agents to early treatment settings, future studies should also focus on the predictive value of TROP-2 expression for TROP-2 targeted ADCs in early and advanced TNBC.

## Supplementary Information

Supplementary Tables.

## Data Availability

The data that support the findings of this study are available upon reasonable request from the corresponding author.

## Abbreviations

ADC: Antibody–drug conjugates
AR: Androgen receptor
BCSS: Breast cancer-specific survival
BMI: Body mass index
CNB: Core needle biopsy
DCIS: Ductal carcinoma in situ
DRFI: Distant recurrence-free interval
EMT: Epithelial-mesenchymal transition
H&E: Hematoxylin and eosin
HER2: Human epithelial growth factor receptor-2
IDFS: Invasive disease-free survival
IHC: Immunohistochemistry
LAR: Luminal androgen receptor
LVI: Lymphovascular invasion
NACT: Neoadjuvant chemotherapy
pCR: Pathological complete response
SG: Sacituzumab govitecan
SMI: Standardized mitotic index
sTILs: Stromal tumor-infiltrating lymphocytes
TNBC: Triple-negative breast cancer
TROP-2: Trophoblast cell-surface antigen-2
