# Supplementary material for: Correlation of TROP-2 expression with clinical–pathological characteristics and outcome in triple-negative breast cancer
Source: Sci Rep. 2022 Dec 28;12:22498. doi: 10.1038/s41598-022-27093-y (PMC9797547; doi:10.1038/s41598-022-27093-y)
Supplement: Supplementary file 1 — Supplementary Tables. [file 41598_2022_27093_MOESM1_ESM.docx]

**Supplementary Table 1**: 2-, 5-, and 10-year IDFS, DRFI and BCSS estimates for all patients.

| **Years** | **% BCSS (95% CI)** | **% DRFI (95% CI)** | **% IDFS (95% CI)** |
| --- | --- | --- | --- |
| 2 | 94.98 (93.02;96.53) | 88.18 (85.46;90.61) | 86.57 (83.58;89.05) |
| 5 | 84.70 (81.62;87.51) | 81.74 (78.52;84.74) | 77.19 (73.58;80.37) |
| 10 | 81.55 (78.20;84.67) | 80.70 (77.40;83.80) | 66.64 (62.42;70.51) |
| BCSS: breast cancer-specific survival, DRFI: distant recurrence-free interval, iDFS: invasive disease-free survival | | | |

**Supplementary Table 2:** Associations of continuous TROP-2 expression with TILs and SMI

| **Variable** | **Category** | **N** | **Median**  **TROP-2 expression H-score** | **IQR**  **TROP-2 expression H-score** | **P-value** |
| --- | --- | --- | --- | --- | --- |
| **sTILs** | **sTILs low** | 367 | 70.0 | (10.0-150.0) | 0.643 |
|  | **sTILs medium** | 106 | 72.5 | (15.0-180.0) |  |
|  | **sTILs high** | 115 | 60.0 | (15.0-150.0) |  |
| **SMI** | **<median** | 224 | 90.0 | (10.0-180.0) | 0.468 |
|  | **>median** | 227 | 60.0 | (20.0-150.0) |  |
| IQR: inter-quartile range; sTILs: stromal tumor-infiltrating lymphocytes; SMI: standardized mitotic index | | | | | |

**Supplementary Table 3:** Associations of categorical TROP-2 expression with categorical clinical-pathological characteristics

|  |  | **Low TROP-2** | **Medium TROP-2** | **TROP-2 High** | **P-value** |
| --- | --- | --- | --- | --- | --- |
| sTILs | N | 342 | 149 | 97 | 0.476 |
|  | Median | 20.5 | 17.0 | 21.0 |  |
|  | IQR | (7.2; 42.0) | (7.0; 42.0) | (8.8; 38.0) |  |
| SMI | N | 257 | 108 | 86 | 0.484 |
|  | Median | 18.0 | 16.2 | 16.0 |  |
|  | IQR | (10.1; 26.7) | (11.0; 23.7) | (10.3; 22.8) |  |
| Age | N | 343 | 149 | 97 | 0.837 |
|  | Median | 52.0 | 54.0 | 55.0 |  |
|  | IQR | (44.0; 67.0) | (47.0; 65.0) | (48.0; 66.0) |  |
| BMI | N | 342 | 148 | 97 | 0.251 |
|  | Median | 24.97 | 24.6 | 24.5 |  |
|  | IQR | (22.6; 28.0) | (22.3; 27.4) | (21.6; 27.3) |  |
| IQR: inter-quartile range; sTILs: stromal tumor-infiltrating lymphocytes; SMI: standardized mitotic index | | | | | |

**Supplementary Table 4:** Associations of categorical TROP-2 expression with categorical clinical-pathological characteristics

| **Variable** | **Statistic** | **low H-score <100** | **medium H-score 100-200** | **high H-score 200-300** | **P-value** |
| --- | --- | --- | --- | --- | --- |
| AR 1% |  |  |  |  |  |
| Negative | n/N (%) | 239/340 ( 70.3%) | 95/148 ( 64.2) | 64/97 ( 66.0%) | 0.369 |
| Positive | n/N (%) | 101/340 ( 29.7%) | 53/148 ( 35.8%) | 33/97 ( 34.0%) |  |
| AR 10% |  |  |  |  |  |
| Negative | n/N (%) | 266/340 ( 78.2%) | 103/148 ( 69.6%) | 70/97 ( 72.2%) | 0.099 |
| Positive | n/N (%) | 74/340 ( 21.8%) | 45/148 ( 30.4%) | 27/97 ( 27.8%) |  |
| sTILS categorical |  |  |  |  |  |
| low (>30) | n/N (%) | 214/342 ( 62.6%) | 96/149 ( 64.4%) | 57/97 ( 58.8%) | 0.552 |
| medium (30-49) | n/N (%) | 57/342 ( 16.7%) | 26/149 ( 17.5%) | 23/97 ( 23.7%) |  |
| high (>50) | n/N (%) | 71/342 ( 20.8%) | 27/149 ( 18.1%) | 17/97 ( 17.5%) |  |
| SMI (binary) |  |  |  |  |  |
| < Median (16.85) | n/N (%) | 118/257 ( 45.9%) | 58/108 ( 53.7%) | 48/86 ( 55.8%) | 0.178 |
| >= Median (16.85) | n/N (%) | 139/257 ( 54.1%) | 50/108 ( 46.3%) | 38/86 ( 44.2%) |  |
| Menopausal status |  |  |  |  |  |
| pre/peri menopause | n/N (%) | 148/338 ( 43.8%) | 56/141 ( 39.7%) | 40/95 ( 42.1%) | 0.711 |
| post menopause | n/N (%) | 190/338 ( 56.2%) | 85/141 ( 60.3%) | 55/95 ( 57.9%) |  |
| BMI categorical |  |  |  |  |  |
| underweight | n/N (%) | 8/339 ( 2.4%) | 4/148 ( 2.7%) | 2/96 ( 2.1%) | 0.792 |
| lean | n/N (%) | 162/339 ( 47.8%) | 79/148 ( 53.4%) | 50/96 ( 52.1%) |  |
| high | n/N (%) | 169/339 ( 49.8%) | 65/148 ( 43.9%) | 44/96 ( 45.8%) |  |
| Tumor size |  |  |  |  |  |
| T1 | n/N (%) | 146/343 ( 42.6%) | 75/149 ( 50.3%) | 45/97 ( 46.4%) | 0.525 |
| T2 | n/N (%) | 159/343 ( 46.4%) | 56/149 ( 37.6%) | 45/97 ( 46.4%) |  |
| T3 | n/N (%) | 23/343 ( 6.7%) | 12/149 ( 8.1%) | 5/97 ( 5.2%) |  |
| T4 | n/N (%) | 15/343 ( 4.3%) | 6/149 ( 4.0%) | 2/97 ( 2.0%) |  |
| N-stage |  |  |  |  |  |
| N0 | n/N (%) | 227/342 ( 66.4%) | 95/148 ( 64.2%) | 54/97 ( 55.7%) | 0.362 |
| N1 | n/N (%) | 85/342 ( 24.9%) | 37/148 ( 25.0%) | 33/97 ( 34.0%) |  |
| N2 | n/N (%) | 18/342 ( 5.3%) | 7/148 ( 4.7%) | 7/97 ( 7.2%) |  |
| N3 | n/N (%) | 12/342 ( 3.4%) | 9/148 ( 6.1%) | 3/97 ( 3.1%) |  |
| AR: androgen receptor; DCIS: ductal carcinoma in situ; LVI: lymphovascular invasion; IBC-NST: invasive breast carcinoma of no special type, sTILs: stromal tumor infiltrating lymphocytes | | | | | |
